# Genome-wide metabolic (re-) annotation of *Kluyveromyces lactis*

**DOI:** 10.1186/1471-2164-13-517

**Published:** 2012-10-01

**Authors:** Oscar Dias, Andreas K Gombert, Eugénio C Ferreira, Isabel Rocha

**Affiliations:** 1 IBB – Institute for Biotechnology and Bioengineering, Centre of Biological Engineering, Universidade do Minho, Campus de Gualtar, 4710-057, Braga, Portugal; 2 Department of Chemical Engineering, University of Sao Paulo, Polytechnic School, PO Box 61548, 05424-970, Sao Paulo, SP, Brazil

**Keywords:** Genome annotation, *Kluyveromyces lactis*, Metabolic functions, Transport systems, *Merlin*

## Abstract

**Background:**

Even before having its genome sequence published in 2004, *Kluyveromyces lactis* had long been considered a model organism for studies in genetics and physiology. Research on *Kluyveromyces lactis* is quite advanced and this yeast species is one of the few with which it is possible to perform formal genetic analysis. Nevertheless, until now, no complete metabolic functional annotation has been performed to the proteins encoded in the *Kluyveromyces lactis* genome.

**Results:**

In this work, a new metabolic genome-wide functional re-annotation of the proteins encoded in the *Kluyveromyces lactis* genome was performed, resulting in the annotation of 1759 genes with metabolic functions, and the development of a methodology supported by *merlin* (software developed in-house). The *new annotation* includes novelties, such as the assignment of transporter superfamily numbers to genes identified as transporter proteins. Thus, the genes annotated with metabolic functions could be exclusively enzymatic (1410 genes), transporter proteins encoding genes (301 genes) or have both metabolic activities (48 genes). The *new annotation* produced by this work largely surpassed the *Kluyveromyces lactis* currently available annotations. A comparison with KEGG’s annotation revealed a match with 844 (~90%) of the genes annotated by KEGG, while adding 850 new gene annotations. Moreover, there are 32 genes with annotations different from KEGG.

**Conclusions:**

The methodology developed throughout this work can be used to re-annotate any yeast or, with a little tweak of the reference organism, the proteins encoded in any sequenced genome. The *new annotation* provided by this study offers basic knowledge which might be useful for the scientific community working on this model yeast, because new functions have been identified for the so-called metabolic genes. Furthermore, it served as the basis for the reconstruction of a compartmentalized, genome-scale metabolic model of *Kluyveromyces lactis*, which is currently being finished.

## Background

The yeast *Kluyveromyces lactis* (*K. lactis*) has long been considered a model organism for studies in genetics and physiology [1]. As pointed out by Fukuhara in 2006 [2], interest in this organism began in academia, mainly due to its ability to metabolize the beta-glycoside lactose and other properties such as its GRAS (generally regarded as safe) status. Biotechnological applications started to be investigated later and, as depicted on the report by van Ooyen *et al*. in 2006 [3], recombinant protein expression has probably been the most widely explored application with *K. lactis*. There are reports that at least two of these proteins, namely prochymosin and lactase (or beta-galactosidase), reached industrial production [3,4].

A common approach used by the scientific community active on *K. lactis* is to either literally work in parallel to or at least in comparison with *Saccharomyces cerevisiae* (*S. cerevisiae*). The Baker’s yeast is not only the best described Eukaryote (it was the first Eukaryote ever to have its genome completely sequenced [5]), but it is also the most employed organism in industry, at least in terms of production volumes.

Energy metabolism is the physiological aspect that mostly distinguishes both species. While the Crabtree-positive yeast *S. cerevisiae* has a strong tendency to ferment, even under aerobic conditions, *K. lactis* is considered Crabtree-negative and preferably uses respiration for energy generation, unless oxygen becomes limiting [6,7]. Another crucial difference between the two yeasts is that *K. lactis*, in contrast to *S. cerevisiae*, is not capable of growing under complete anaerobiosis [8].

Research on *K. lactis* (a.k.a. milk yeast) is quite advanced and includes aspects such as the glucose sensing and repression cascade [9,10], the molecular basis for the Crabtree-negative characteristic of this yeast [11], the improvement of secretory pathways for heterologous protein expression [12,13], the engineering of post-translational modifications with the aim of avoiding hypermannosilation of heterologous proteins [14], the oxidative stress response [15,16], the molecular basis for the incapacity of growing anaerobically [8,17], the description of its transcriptional regulators [18], and an exhaustive study of its cell wall [19]. Remarkably, many of the physiological differences between *K. lactis* and *S. cerevisiae* seem related to the whole-genome duplication event [20], which affected *S. cerevisiae*, but not *K. lactis*.

One of the key aspects of research on *K. lactis* is the fact that most of the work performed in the past decades has been based on a single strain, namely CBS 2359 (a.k.a. NRRL Y-1140). This has facilitated enormously the interpretation of results and the interaction among laboratories throughout the world [2]. Another important factor is that, in spite of all historical changes in terms of taxonomic methods, mainly the recent adoption of criteria purely based on gene sequences, *K. lactis* remains *K. lactis*, even after a recent redefinition of the *Kluyveromyces* and related genera [21,22].

*K. lactis* is one of the few yeast species with which it is possible to perform formal genetic analysis [2]. Additionally, due to some recent advances [19,23,24], molecular tools have been developed, facilitating the generation of mutants [1], a task which can now be considered as simple to perform with this yeast as it is with *S. cerevisiae*. Also, its full genome sequence was made available some years ago [25], allowing for the improvement of our understanding on eukaryotic genome evolution by comparing the genomes of different yeast species. Within this context, a number of works have been published on particular aspects of yeast genomes [26-33].

### (Re-)Annotation

There are several reasons to re-annotate a genome, such as: new genes or protein functions being discovered, a research group trying to determine the reproducibility of an existing annotation, or just because the information associated to a specific organism is known to be out-dated. Thus, the re-annotation of a genome, especially for genes classified as hypothetical proteins, is very important for assuring an up-to-date gene annotation and not compromising future similarity alignments for newly sequenced genes.

Functional annotation can be defined as the inference and assignment of functions to genes or proteins. Such information is often obtained by similarity to formerly characterized sequences, found in several online or local databases [34]. Likewise, the re-annotation process can be depicted as the annotation of a previously annotated gene or full genome [34,35].

Though being uncommon, there are some examples of genome wide re-annotations, such as *Campylobacter jejuni* NCTC11168 [35]*Mycobacterium tuberculosis* H37Rv [36], and *Arabidopsis thaliana*[37]. All of the above annotations assigned new functions to genes that had been previously identified as “hypothetical proteins” and corrected some of the previous annotations.

A genome-wide metabolic functional annotation is a thorough effort which has the objective of trying to determine and label the genes involved in the metabolism of the organism of interest, skipping the regulatory and other genes annotation. Therefore, only the genes that encode enzymes or transporter proteins will be assigned with a function and included in this re-annotation.

*Kluyveromyces lactis* genome does not have an official genome-wide functional metabolic or other annotation in the GenBank [38] and Reference Sequences (RefSeq) [39] databases ( http://www.ncbi.nlm.nih.gov/sites/entrez?Db=genome&Cmd=ShowDetailView&TermToSearch=17850). The annotation available in GenBank files ( ftp://ftp.ncbi.nih.gov/genbank/genomes/Fungi/Kluyveromyces_lactis_NRRL_Y-1140_uid12377 any *.gbk) in the GenBank database only characterizes the gene products by applying the same code used for the gene identification, followed by a “p” instead of a “g”; for instance, /locus_tag=”KLLA0A00132g” was assigned with /product = "KLLA0A00132p". On the other hand, RefSeq ( ftp://ftp.ncbi.nih.gov/genomes/Fungi/Kluyveromyces_lactis_NRRL_Y-1140_uid12377/ any *.gbk) database assigns all proteins as hypothetical proteins. Nevertheless, all genes have descriptions in the GenBank “\notes” field. For example, the KLLA0A08492g gene is described as encoding a "conserved hypothetical protein", the KLLA0A08536g gene has "some similarities with uniprot|P25587 *Saccharomyces cerevisiae* YCL005W" and the KLLA0A08624g gene is "highly similar to uniprot|Q75ET0 *Ashbya gossypii* AAL002W AAL002Wp and similar to YCL001W uniprot|P25560 *Saccharomyces cerevisiae* YCL001W RER1 Protein…”. Other genes have more explicit annotations, for instance gene KLLA0A00891g is described as "uniprot|P53768 *Kluyveromyces lactis* KLLA0A00891g HAP2 Transcriptional activator HAP2", KLLA0F13530g is a "uniprot|P49385 Kluyveromyces lactis ADH4 Alcohol dehydrogenase IV, mitochondrial precursor" and KLLA0D00231g is described as "uniprot|Q9Y844 Kluyveromyces lactis mal22 Maltase" (in agreement with the new annotation). However, these descriptions are not considered annotations, because relevant information, such as the gene product and, when available, the Enzyme Commission (EC) number [40], is not provided in most cases. Furthermore, when available, such information should be delivered in the correct GenBank field (“/product” and “/EC number” instead of the “/notes” field) for easier manipulation using bioinformatics tools and user appraisal. Other databases such as KEGG ( http://www.genome.jp/kegg/kegg2.html) [41] perform metabolic annotations, with fairly acceptable results, though failing in some annotations and missing several genes with metabolic functions. (Universal Protein Resource) UniProt ( http://www.ebi.ac.uk/UniProt/), on its hand, is composed by two databases, Swiss-Prot and TrEMBL, which are curated and non-curated, respectively [42]. The curated database provides information that was manually annotated and reviewed, even if it was obtained electronically. Such database contains some information about the microorganism studied during this work, though somewhat scarce.

Hence, in this work we propose a genome-wide metabolic (re-)annotation of the proteins encoded in the *Kluyveromyces lactis* complete sequenced genome, identifying the genes involved in metabolites conversion and carriage throughout the cell, which is imperative for the reconstruction of a robust genome-scale metabolic model.

### Genome-scale reconstructed metabolic models

Full genome sequences have been used, among many other applications, to reconstruct metabolic networks of different microorganisms such as *Escherichia coli*[43] or *Saccharomyces cerevisiae*[44]. This allows for the establishment of the so-called genome-scale metabolic models, which are developed bottom-up from the genome up to the reactions catalysed by the enzymes encoded in such set of genes. It is an iterative process that culminates in a reaction set that is used to simulate *in silico* the phenotype of the studied organism, under several environmental or genetic conditions [45]. The use of such models has resulted in insight gaining and hypothesis testing, such as the enhancement of sesquiterpene production in *Saccharomyces cerevisiae*[46], the improvement of the production of succinic acid in *Escherichia coli*[47] or finding new targets in drug research [48].

For the reconstruction of a robust genome-scale model, it is mandatory to have a proper annotation of the genome. For a metabolic model, all genes with metabolic roles, such as enzymes and transporters, have to be identified. The reconstruction of a metabolic model is a laborious and extensive process that has been described by Thiele and Palsson in 2010 [49] as a 96 steps protocol, which takes a long time to be completed, depending on data availability. Such work also describes the first step “*1| Obtain genome annotation*” as a critical step, thus the importance of a robust annotation for the reconstruction process.

Although the genome of *K. lactis* has been publicly available for some years, a complete functional annotation was not made available to the public yet. In 2009, Souciet *et al*. [33] re-annotated the genome of *K. lactis*, together with the sequencing and annotation of other yeast genomes, with the aim of performing comparative genomics. However, such annotation did not propose a functional annotation for each *K. lactis* gene. Here we present a work which identifies genes with metabolic functions and assigns functions to those genes, such as EC numbers, Transporter Classification Superfamily (TCS) numbers and Transporter Classification (TC) numbers [50]. Whenever a complete EC number (‘class’.’subclass’.’sub-subclass’.’enzyme serial number’) was not available, a partial EC number was assigned to such enzymes (‘class’.’subclass’.’sub-subclass.-, ‘class’.’subclass’.-.- and ‘class’.-.-.-).

The re-annotation of the proteins encoded in the *K. lactis* CBS 2359 metabolic genome was performed in a semi-automatic manner by combining the use of the software *merlin*[51], developed in-house and available for download (at http://www.merlin-sysbio.org) and manual inspection. The annotated genome of this organism brings some new insights on its capabilities and allowed the reconstruction of the *Kluyveromyces lactis* genome-scale metabolic model (currently being finalized). *merlin*’s dynamic annotation tool was used to perform first an automatic re-annotation of the complete genome followed by a manual curation of the enzymatic annotation. *merlin*’s transporter annotation tool was used to identify genes that encode transporter proteins, as well as the metabolites transported by such systems. In the end, a new, re-annotated, GenBank file was created by *merlin* for each *K. lactis* chromosome.

We believe that this re-annotation not only served as the basis for the assembly of a genome-scale metabolic model for *K. lactis,* but also provides relevant biological information for the scientific community dealing with this organism and yeasts in general.

## Methods

### Online databases

Several online databases were used throughout this work. A brief description of each one is available bellow:

• The first Basic Local Alignment Search Tool (BLAST) [52] similarity search performed with *merlin* used All non-redundant sequences (including GenBank coding sequences translations, RefSeq Proteins, Brookhaven Protein Data Bank (PDB), SwissProt, Protein Information Resource (PIR), Protein Research Foundation (PRF) databases) (*nrDB*) available in the National Center for Biotechnology Information (NCBI) databases [39] to find any protein sequence similar to translated *K. lactis* genes.

• A second BLAST search used NCBI’s yeast database (*yeastDB*) [39], which is a single curated set of *Saccharomyces cerevisiae* protein sequences available at the NCBI's RefSeq database.

• The Entrez Protein ( http://www.ncbi.nlm.nih.gov/sites/entrez?db=protein) database is a collection of sequences from several sources, including GenBank CDS translations, RefSeq Proteins, SwissProt, PIR, PRF, and PDB [39]. *Entrez Protein* provided all information that *merlin* retrieved for each *Kluyveromyces lactis* homologue gene.

• The UniProtKB/Swiss-Prot ( http://www.UniProt.org/) database is a manually curated protein sequences database which provides annotations with minimal redundancy and high level of integration with other databases [42]. Thus, UniProtKB/Swiss-Prot was selected as a reference resource during the *Kluyveromyces lactis* genomic re-annotation.

• The Saccharomyces Genome Database (SGD – http://www.yeastgenome.org/) project collects information and maintains a database of the molecular biology of the yeast *Saccharomyces cerevisiae*[53]. This database includes a variety of genomic and biological information and is maintained and updated by curators. The SGD was selected as the second reference database for this project.

• The Comprehensive Enzyme Information System BRaunschweig ENzyme DAtabase (BRENDA – http://www.brenda-enzymes.info/) provides enzyme functional data obtained directly from literature by professional curators [54]. This database was used to confirm the information gathered in the previous two databases, thus being the third reference database selected for this work.

• The Transporter Classification Database (TCDB – http://www.tcdb.org/) details a comprehensive classification system, approved by the International Union of Biochemistry and Molecular Biology (IUBMB), for membrane transporter proteins known as the Transporter Classification (TC) system. The TC system is analogous to the Enzyme Commission system for classification of enzymes, except that it incorporates both functional and phylogenetic information [55]. This database was selected to annotate transporter proteins.

### MEtabolic models reconstruction using genome-scaLe INformation (*merlin*)

*merlin*[51] is a software tool, in continuous development, created to assist on the process of reconstructing a genome-scale metabolic model. The reconstruction process cannot begin without a functionally annotated genome; thus, *merlin* performs automatic genome-wide functional (re)annotations, by comparing biological sequences from the organism being studied with all of the NCBI’s databases. *merlin* provides a numeric confidence score for each automatic assignment, taking into account the frequency and the taxonomy within the annotations of all sequences that are similar to the gene under investigation [51], according to equation (1):

(1) $${\text{score}}_{\text{annotation}}=\alpha \cdot {\text{score}}_{\text{frequency}}+\left(1-\alpha \right)\cdot {\text{score}}_{\mathrm{taxonomy}}$$

In which the frequency score is related with the number of times a given function (EC number) appears in the set of homologues and the taxonomy score is related with the taxonomic proximity between the studied organism and those in which those functions had been identified. The user can choose to give more relevance to the frequency score or to the taxonomy score, just by altering the alpha value in *merlin*’s interface (see Additional file 1: Figure S1 of the supplemental material). If the user considers the frequency more relevant than the taxonomy of the homologue genes the alpha value should be set between 0.5 and 1. If taxonomy is preferred over frequency the value should be between 0 and 0.5. In this work, the α value was set to 0.2, so that the yeasts’ annotations could be given more relevance than other organisms’ annotations.

However, in this work *merlin*'s automatic annotation was fully reviewed to maximize the re-annotation confidence.

Moreover, *merlin*’s interface was used throughout the (re)annotation process to assign functions and protein names to each metabolic gene. *merlin*’s interface is particularly user friendly, providing “drop down boxes” (see Additional file 1: Figure S1 of the supplemental material) for the annotation of each gene. *merlin* allows exporting the annotation as an Excel file or in the GenBank format, during or after the end of the annotation process.

### Identification of genes that encode enzymes

To retrieve enzymatic information, *merlin* performs remote BLAST similarity searches to the NCBI databases. When the purpose of performing BLAST similarity searches is to retrieve metabolic information for a genome re-annotation, the output of a BLAST similarity search can be too minimalistic and very confusing. Anyone that has tried one of the many BLAST search tools available in the internet (such as http://blast.ncbi.nlm.nih.gov/Blast.cgi or http://www.UniProt.org/blast/) knows that the output of a BLAST search is not much helpful for the collection of metabolic data (see Additional file 1: Figure S2 of the supplemental material), because the user has to follow several links to retrieve the data: to retrieve metabolic data, the user has to go over all identified homologue genes, retrieve enzymatic information and compile such information for all genes of the studied genome. To avoid such massive effort, *merlin* was used to implement the remote similarity alignments between the user set of genes (or full genome as was the case) and the previously selected remote NCBI database, as well as retrieve and classify each homologue’s annotation, providing comprehensible information.

**Figure 1:**
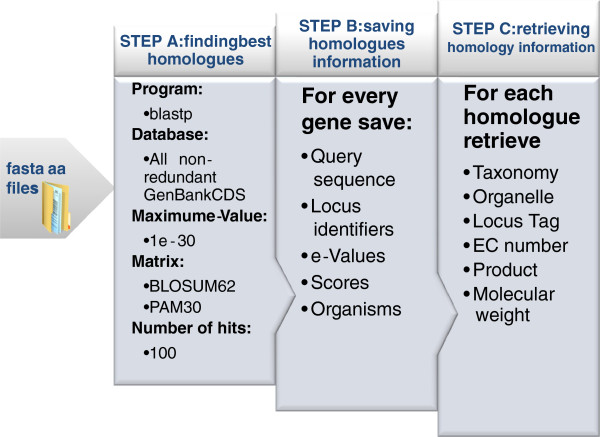
***merlin*’s path from organism genome to enzymatic homology data.** The BLAST search (configured with the parameters presented above) was performed in the first stage of the homology data inference (STEP A). Specific information for each gene homologues, such as identifiers or scores, is parsed and saved in STEP B. Finally, the *Entrez protein* web services are used to retrieve the metabolic information, such as EC numbers or taxonomy in STEP C.

The path from genome sequence information to enzymatic data retrieved from homology is described in Figure 1. Initially, *merlin* received the *K. lactis* genome in the amino acid fasta format, downloaded from the GenBank repository at ftp://ftp.ncbi.nih.gov/genomes/Fungi/Kluyveromyces_lactis_NRRL_Y-1140_uid12377/.

Then *merlin* performed the remote BLAST similarities search, configuring the algorithm with the parameters also depicted in the first step of the figure. At the time of the similarity search (January 2010) the *nrDB* was a collection of 10,140,583 sequences and the *yeastDB* encompassed 6298 sequences.

The program used to perform the remote blast search was the blastp (version 2.2.22+ at the time of the BLAST). The e-value is used to create a significance threshold for returning results. A lower e-value will result in a shorter list with more quality homologues, thus the maximum e-value threshold was set to 1E-30.

The matrices referred in Figure 1, are parameters of the BLAST algorithm, and are used to evaluate the quality of a pairwise sequence alignment by assigning scores for the alignment of any possible pair of residues. BLOSUM 62 was used as the default matrix for the similarity search algorithm configuration and was changed to PAM30 for the shorter sequences that could not be aligned with the first matrix *merlin* takes approximately 24 h to automatically assign a functional annotation to every protein encoded in a given genome, depending on the NCBI servers’ availability and the genome size.

For each *Kluyveromyces lactis* gene, the top 100 most similar homologues were retrieved and the information displayed in Figure 1 – Step 2 was collected. If less than 100 homologues were available, only those were processed. Afterwards, *merlin* accessed the *Entrez Protein* webservice to download and save several data for each homologue acquired in the previous step. Such data is listed in Figure 1 – Step 3.

Using internal heuristics, described in [51] and briefly represented above in equation 1, *merlin* automatically selected a candidate annotation for each protein encoding gene of the studied genome based on confidence scores. The similarity result (gene product, EC number) with the highest confidence score was selected by *merlin* to automatically annotate each protein encoding gene of the studied genome. Moreover, *merlin* reduced the curation efforts, as it allows the user to browse through all similarity search results and change the automatic annotations provided by the software.

When the first automatic annotation results were analysed, a pattern emerged. The homologues’ taxonomic distribution was, as it will be shown in the Results section, biased. Indeed, whenever a *Saccharomyces cerevisiae* homologue was available, *merlin* would consistently select the baker’s yeast gene annotation to annotate the *Kluyveromyces lactis* gene. Thus, the baker’s yeast was selected as a reference organism for the EC numbers annotation because the two microorganisms share the phylogenetic lineage all the way to the taxonomic family level and *S. cerevisiae* is the best studied, annotated and curated Fungus. Hence, two projects were initiated with *merlin*, allowing the software tool to use all data available in the NCBI database (*nrDB*) to annotate the *Kluyveromyces lactis* genome in the first project, while for the later project only data from the NCBI’s *yeastDB* were used. Each *K. lactis* gene assigned by *merlin* with enzymatic functions on either the first or the second similarity search was labelled as an enzyme encoding gene candidate (EEGC).

**Figure 2:**
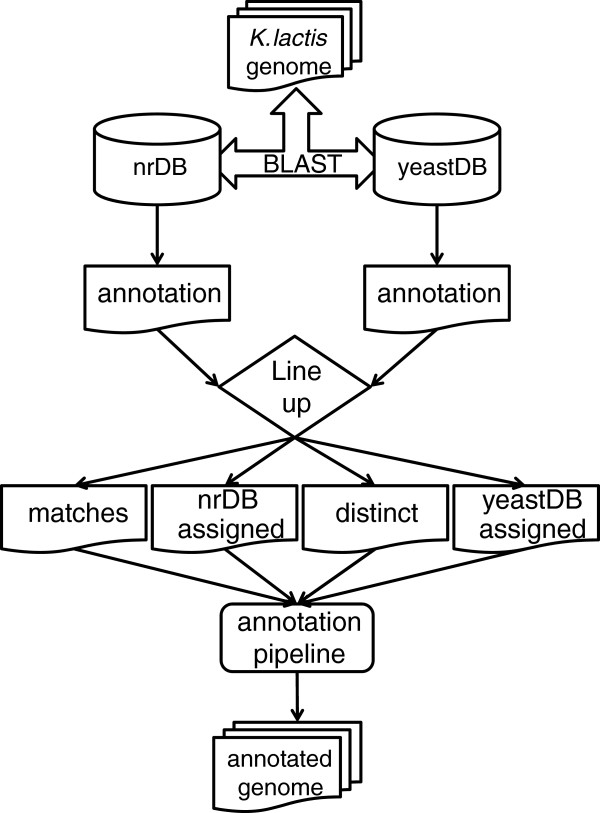
**Enzymes annotation scheme.** BLAST searches were performed to a pair of distinct databases (*nrDB* and *yeastDB*), originating two parallel annotations. Four sets of genes were assembled from the comparison of such annotations: the group of genes with the same assignments in both annotation projects (matches), the genes with different assignments in each project (distinct) and two groups with the genes only annotated in either the *nrDB* or in the *yeastDB*.

**Figure 3:**
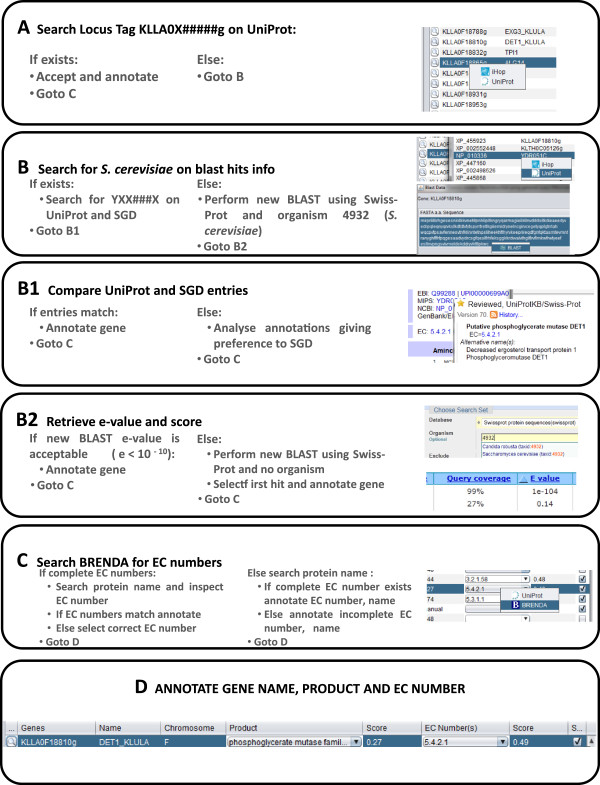
**Annotation pipeline for the assignment of enzymatic functions to *K. lactis *genes.** Each EEGC locus tag was firstly queried on UniProt and, if present, the assignment was accepted and the gene was annotated. If not, then a *S. cerevisiae* gene was sought in the BLAST hits kept by merlin for such gene (STEP B). If a baker’s yeast homologue (STEP B1) was available, its identifier (YXX####x) was searched in both UniProt and SGD databases. When both databases records were identical, the gene was annotated; else, the records would be examined and the SGD entries would be favoured. For the EEGC’s that did not have any *S. cerevisiae* homologue (STEP B2), a new specific similarity search was performed in NCBI BLAST, restraining the possible outcomes to Swiss-Prot reviewed records and the organism to *S. cerevisiae,* with the acceptable e-value decreased to e < 1E-10. If there was an entry that complied with those conditions, the gene was annotated; else, the BLAST similarity search was unrestricted, organism wise. Again, if there was an entry that complied with the previous conditions, the gene was annotated as homologue of the first hit, else it was discarded. The previously annotated information was revised in BRENDA to verify the function about to be annotated to such gene (STEP C). Finally, the information collected in the previous steps is assigned to the EEGC (STEP D), rendering the EEGC a metabolic gene or discarding such gene as metabolic.

The developed approach originated two parallel annotations, as depicted in Figure 2, which allowed comparing the functional assignments for each gene. From this line up, four sets of genes were assembled. The EEGC’s assigned with the same enzyme by both projects were labelled as matches. Such genes’ annotation was generally accepted (although reviewed according to Figure 3), except for partial EC numbers (which were revised on behalf of the existence of complete EC numbers) and deprecated EC numbers (which were updated).

The second set encompassed those genes which were identified as EEGC’s on the first BLAST search (*nrDB* assigned) but not on the second similarity alignment. Such set presented a high number of genes that, although being automatically annotated with metabolic functions, were later discarded by the annotation pipeline depicted in Figure 3 (false positives).

The third set of genes was the most troublesome. It was the group of genes assigned with different enzymes on each *merlin* project (distinct). Such collection was carefully reviewed, with the purpose of selecting the correct gene function without reservations.

The last set (*yeastDB* assigned) encompassed milk yeast EEGC’s which were not automatically annotated as enzymes by *merlin* in the first alignment, but when the search was performed against NCBI’s *yeastDB,* at least one *Saccharomyces cerevisiae* metabolic homologue was identified for each *K. lactis* gene. *merlin* did not assign any annotation on the first similarity search probably because each of those *K. lactis* EEGC had more than 100 homologues in organisms other than *S. cerevisiae* on such alignment.

The EEGC’s were manually verified by following several confirmation steps as depicted in the functional annotation pipeline (Figure 3). The described methodology can be recurrently executed, re-annotating a given genome whenever the user wants to, taking advantage of the up to date information available in NCBI remote BLAST databases.

### Annotation pipeline

Despite using *merlin*, all of the *Kluyveromyces lactis* functional EEGC’s automatic assignments were reviewed according to the schema depicted in Figure 3, so that the minimum number of false positives would be included in this annotation. For that purpose, the main criteria were, in first priority, the existence of information in curated databases for the *K. lactis* genes and, in second priority, the existence of curated *S. cerevisiae* homologues. Only when none of the previous information was available the search was extended to curated homologues of other organisms.

Initially, for each EEGC, a query was performed in UniProt, using the gene locus identifier (locus tag), to assess the existence of a reviewed annotated record for such gene. If UniProt had already identified such gene’s product on a reviewed record, or any literature was available and confirmed the proposed gene annotation, the assignment was accepted and the gene was annotated (after EC number confirmation in BRENDA – Figure 3-C).

On the other hand, if UniProt had no reviewed match for such gene, then a *S. cerevisiae* gene was sought in the BLAST hits (Figure 3-B) kept by *merlin* for such gene. So, if a baker’s yeast homologue was available, its identifier (YXX####x) was searched in both UniProt and SGD databases. After the analysis of the UniProtKB/Swiss-Prot and the SGD entries two situations could arise (Figure 3-B1): the records could be either identical or distinct. When identical, the gene was annotated; else, the records would be thoroughly examined and the SGD entries would be always favoured. As explained above, both UniProtKB/Swiss-Prot and SGD are manually curated databases, thus both results are reliable. Nevertheless, the SGD is favoured when a conflict arises between both databases because it is specific for *Saccharomyces cerevisiae*, and consequently the curators of this database are specialized in the analysis of the baker’s yeast genome. Hence, if the similarity between the *K. lactis* and the *S. cerevisiae* gene sequences is acceptable (e- value < 1E-30) the *K. lactis* gene is considered homologous to the baker’s yeast one and the first is assigned with the same function as the latter.

For the EEGC’s that did not have any *S. cerevisiae* homologue (Figure 3-B2), a specific similarity search was performed in the NCBI BLAST web interface, restraining the possible outcomes to Swiss-Prot reviewed records and the organism to the 4932 taxID (*Saccharomyces cerevisiae*). This step was performed because *merlin*’s scorer was configured to calculate the function scores using the first 100 homologues retrieved from the BLAST similarity search. However, the *S. cerevisiae* homologue could have a cardinality of more than 100. When performing this specific homology search, the number of hits is considerably reduced, thus the acceptable e-value is also decreased to e < 1E-10. If there was an entry that complied with the previous conditions, the gene was annotated; else, the BLAST similarity search was unrestricted, organism wise. Again, if there was an entry that complied with the previous conditions, the gene was annotated as homologue of the first hit, else it was discarded.

Whatever was the source of the candidate enzyme assigned to a given gene, such information was revised in BRENDA to verify the function about to be annotated to such gene (Figure 3-C). Some of the enzymes encoded in the genome were assigned with partial EC numbers by the studied databases. BRENDA was also used to try to identify complete EC numbers for such genes, by searching for the names of those gene products in that database.

Finally, the information collected in the previous steps is assigned to the EEGC, as depicted in Figure 3-D, rendering the EEGC a metabolic gene or discarding such gene as metabolic.

### Classification of manual curation results

When using the annotation pipeline to analyse the EEGC’s, a limited number of logical jumps were detected. Therefore, an alpha-numeric cross classification system was developed to log and identify the gene classification patterns, encompassing the origin of the entry chosen in the final annotation (*nrDB* or *yeastDB*) and the database(s) that provided the information that motivated the choice made. A detailed description of such classification is available in Additional file 2 of the supplemental material.

### Identification of genes that encode transporter proteins

Only four *Kluyveromyces lactis*’ genes are available in TCDB as transporter protein encoding genes (see Additional file 3: Table S1 of the supplemental material). Therefore, it was necessary to implement a methodology to further identify transporter proteins using homology analysis.

Although *merlin* uses remote BLAST similarity searches to classify gene products, the transporter information is obtained by performing local smith-waterman (SW) similarity alignments [56] with the TCDB, to identify the TCS (Transporter Classification Superfamily) number of the genes that encode transporter proteins. This methodology was also developed in-house and will be included in *merlin’s* 2.0 version. An article with the detailed description of this methodology (Genome-wide semi-automated annotation of transporter systems, Dias *et al.*, 2012) has been recently submitted.

Unlike enzymes, transporter proteins cannot be directly classified from homology. Enzymes are represented by EC numbers that classify the catalysed reactions and a gene can be annotated with several EC numbers. TC numbers are associated to proteins that transport a specific range of substrates and are often associated to a single gene. For example, a gene that encodes a carrier that is able to transport a range of substrates is assigned with a single TC number and not a range of TC numbers, as is the case with EC numbers. TC numbers are grouped in TC families. For example, the 2.A.1.1 – The Sugar Porter (SP) Family encompasses transport proteins that transport sugars. Likewise, TC families are grouped in TCS. For example, the 2.A.1–The Major Facilitator Superfamily (MFS) includes the 2.A.1.1. The Sugar Porter (SP) Family, the 2.A.1.2 – The Drug:H + Antiporter Family and several other families. Therefore, for the classification of the genes that encode transporter proteins, the approach was somewhat different and is concisely described next.

The process of performing genome-wide similarity searches using the SW algorithm, despite being more accurate than BLAST, can be very time–consuming, as such alignments are very demanding. Therefore, the number of *K. lactis* genes aligned against TCDB was reduced via the *TransMembrane prediction using Hidden Markov Models* (TMHMM) [57] software. TMHMM is a prediction algorithm that identifies the number of transmembrane helices in a protein using hidden Markov models.

Thus, all genes that had one or more transmembrane helices were considered transporter protein encoding gene candidates (TPGC) and were aligned to the TCDB. The similarity threshold, when performing the SW similarity searches, was of 10%, because the transporter database was very small (6100 records at the time of the alignment – September 2011). Moreover, *merlin* uses internal heuristics to lower the threshold, inversely to the number of transmembrane helices of the gene.

A TPGC can have similarities to different families and super-families of the same TC class that can nevertheless have similar functions. Thus, the TC family numbers, as well as the metabolites, of the TCDB genes similar to each TPGC were classified with the same algorithm used by *merlin* to classify the EC numbers of each EEGC. Such algorithm classified the TC family numbers and metabolites associated to each TPGC, using the taxonomy of each of the TCDB homologue genes and the frequency of the TC family numbers or metabolites, within all similar genes. In the end of this process, each gene identified as a TPGC was either discarded (not considered a transporter protein) or effectively annotated as a transporter protein encoding gene. In the latter case, a TCS number, as well as the metabolites transported by such protein, were assigned to each transporter protein encoding gene. Since it was considered that the transporter family number could be too restrictive, it was decided to go up a level and the TCS number was chosen instead.

## Results and discussion

### Genes annotation

**Figure 4:**
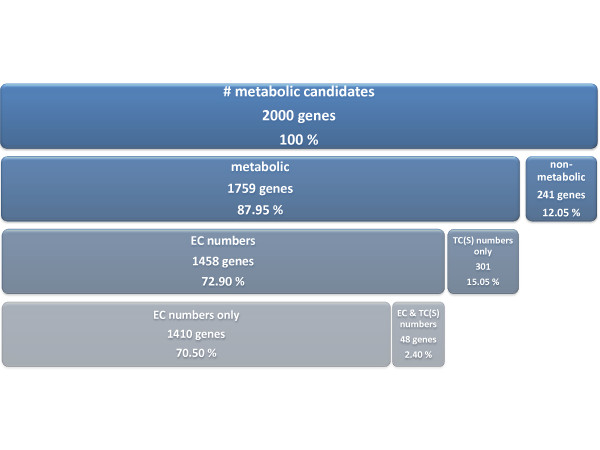
**Annotation statistics.** The level of detail increases downwards

The proteins encoded in the *Kluyveromyces lactis* complete metabolic genome were annotated, systematically, throughout this work. Figure 4 discretises the main outcomes of this process. Out of the 5085 genes available on the GenBank fasta files provided to *merlin*, 2000 genes were revised.

The annotation pipeline for genes that encode enzymes (described on Figure 3 of the Methods section) reviewed a total of 1699 EEGC’s and the transporter annotation function within *merlin* provided 349 genes. However, 48 genes identified as transporter systems encoding genes were also annotated by the annotation pipeline with EC numbers. Hence, such genes were annotated with both transport (TCS or TC numbers) and reaction facilitation (EC numbers) activities.

The annotation pipeline ruled out 241 *K. lactis* EEGC’s as non-metabolic genes because the implemented routine suggested that such EEGC’s homologues were either wrongly assigned as similar to *K. lactis* or incorrectly annotated. The other 1458 genes were confirmed and annotated as metabolic genes.

As depicted in Figure 4, most of those 1458 genes were annotated with at least one EC number and 301 were annotated as exclusively transporters, being assigned with TCS or TC numbers. Summing up, 1759 genes were classified as metabolic genes, of which 1410 are exclusively enzymatic, 301 exclusively transporter proteins and 48 have both functions.

The final annotation of each EEGC is available in Additional file 3: Table S2 of the supplemental material.

The *Kluyveromyces lactis* genome had been sequenced by the Génolevures consortium; however, the genes identified by the consortium were not assigned with EC or TC numbers. Also, despite holding the genome sequencing data, GenBank does not provide any functional annotation. Thus, the *new annotation* provided by this work was compared with the data available in KEGG, UniProt and to a lesser extent with BRENDA and TCDB.

The *new annotation* produced by this work largely surpassed the *Kluyveromyces lactis* currently available annotations, as demonstrated in Table 1.

**Table 1:** Comparison of the results reached in this work and previous annotations available

	**KEGG annotation**	**UniProt annotation**	**TCDB annotation**	**BRENDA EC #**	***New annotation***
**# of genes**	938	354	4	34* (38 )**	1759

Number of *K. lactis* genes annotated in each database and the *new annotation*. *BRENDA provides the number of enzymes associated to *K. lactis*.

**In brackets the total number of EC numbers, including four EC numbers incorrectly associated to *K. lactis* on BRENDA.

### Comparison with KEGG

The comparison between the *new annotation* and KEGG’s annotation is depicted in Additional file 3: Table S3 of the supplemental material. The *new annotation* matched 844 (~90%) of genes annotated by KEGG, adding 850 new gene annotations. Moreover, there are 32 genes with annotations different from KEGG.

Also, 19 genes were assigned with more enzymes on the present annotation than on the KEGG annotation. For instance, KEGG annotates the KLLA0B02717g gene with the EC number 2.3.1.86. However, our *new annotation* assigns 6 EC numbers (2.3.1.86, 4.2.1.61, 1.3.1.9, 2.3.1.38, 2.3.1.39, 3.1.2.14) to such gene; thus, KEGG’s annotation is not incorrect but it is a subset of the present study’s annotation. On the other hand, there were 9 genes that were assigned with more enzymes on KEGG than on the present annotation. Finally, the annotation pipeline ruled out 29 genes annotated as metabolic on KEGG, due to several reasons. For instance, KEGG assigns the EC number 2.7.7.7 to KLLA0C11341g, and the *new annotation* identified such gene as an “Accessory subunit of DNA polymerase zeta” with no catalytic activity. KEGG assigns the EC number 6.3.2.19 to KLLA0C08041g. However, the *new annotation* identified that gene as a general negative regulator of transcription. These two, along with 27 other ruled out genes are described in Additional file 3: Table S4 of the supplemental material.

Table 2 contains all genes for which KEGG assigns more enzymes than the *new annotation*. The 1^st^ and the 2^nd^ genes in the table were assigned with one EC number on the *new annotation* because the *S. cerevisiae* homologue only encodes one EC number. The other EC number assigned by KEGG is for a different protein (asparaginyl-tRNA synthase), thus being excluded by the annotation pipeline. The 3^rd^ to the 8^th^ genes in Table 2 were also annotated with only one EC number given the baker’s yeast homologue annotations, verified on SGD and UniProt. Finally, according to KEGG, KLLA0E19625g is associated both with Glutamate synthase [NADH], and Glutamate synthase [NADPH], because UniProt and SGD disagree, the annotation pipeline was followed and only the second EC number was chosen.

**Table 2:** ***New annotation versus *** **KEGG annotation**

**Gene**	** KEGG**	***New annotation***	***S. cerevisiae *** **homologue**	**Protein**
KLLA0A09845g	6.3.5.6, 6.3.5.7	6.3.5.7	YMR293C	glutamyl-tRNA(Gln) amidotransferase
KLLA0E20659g	6.3.5.6, 6.3.5.7	6.3.5.7	YBL080C	glutamyl-tRNA(Gln) amidotransferase
KLLA0B07513g	2.7.1.105, 3.1.3.46	3.1.3.46	YJL155C	fructose-2,6-bisphosphatase
KLLA0E07173g	4.2.1.51, 5.4.99.5	4.2.1.51	YNL316C	prephenate dehydratase
KLLA0E10143g	3.1.3.12, 2.4.1.15	3.1.3.12	YDR074W	trehalose-phosphatase
KLLA0F20548g	2.6.1.19, 2.6.1.22	2.6.1.19	YGR019W	4-aminobutyrate aminotransferase
KLLA0E17997g	3.1.3.16, 3.1.3.48	3.1.3.48	YIR026C	tyrosine-protein phosphatase
KLLA0C09966g	2.8.1.1, 2.8.1.2	2.8.1.1	YOR251C	thiosulfate sulfurtransferase
KLLA0E19625g	1.4.1.14, 1.4.1.13	1.4.1.14	YDL171C	glutamate synthase [NADH]

Cases in which KEGG assigns more EC numbers than the *new annotation*.

Since KEGG’s annotation does not provide any transporter information, whenever a gene encoded a protein with both EC and TC(S) numbers, the transport system was ignored in the assessment, which helped to raise the number of matches between both annotations.

The functions only provided by the *new annotation*, when compared to KEGG, are distributed as described in Table 3. The *new annotation* provides 850 genes of which 524 are enzyme encoding genes not available in KEGG and 326 are associated with transport reactions.

**Table 3:** Summary of genes not available on KEGG’s annotation but annotated in this work

**Genes not annotated in KEGG**		**Number of genes**
complete	EC numbers	318
	EC numbers + TC(S) numbers	23
partial	EC numbers	206
	EC numbers + TC(S) numbers	2
TC + TCS		301

Complete EC numbers are assigned when all classes are identified (*e.g.* 1.1.1.1). Partial EC numbers are assigned when at least one subclass is unknown (*e.g.* 1.1.-.- or 1.1.1.-).

### Comparison with UniProt

All 354 genes annotated with enzymatic functions by UniProt were included in the present annotation by the annotation pipeline, as described in Additional file 3: Table S5 of the supplemental material. For some (48) of those genes more information was collected, either by adding more enzymatic functions (e.g. KLLA0E01959g was annotated with 2.5.1.9 by UniProt and with 2.5.1.9 and 2.5.1.78 in the *new annotation*) or just by providing a complete EC number to a partial UniProt annotation (e.g. KLLA0B01265g is annotated with 3.2.2.- in UniProt and with 3.2.2.27 in the *new annotation*).

### Comparison with BRENDA

BRENDA’s annotation assessment was somewhat different from the other annotations evaluation, as BRENDA does not provide gene information. Hence, the EC numbers provided by BRENDA were sought in the *new annotation* to confirm if there was at least one gene that encoded such enzyme. The *new annotation* included all 34 EC numbers assigned by BRENDA to *K. lactis*, as depicted in Additional file 3: Table S6 of the supplemental material. However, there were 4 other EC numbers associated to *K. lactis* on BRENDA that were not found in the *new annotation*. One of those EC numbers (1.4.1.15) was associated to *K. lactis* because it has an annotation declaring that there is “no activity in *Kluyveromyces lactis*”. Another one of those EC numbers was from a *plasmid*[58] and the other two were from vectors inserted in a *K. lactis* strain to test the viability of the organism as a recombinant protein producer [59].

### Homologues taxonomic distribution

Translated genomes of different organisms were used as reference when performing the homology-based genomic annotation. Thus, an analysis of the phylogenetic distribution of those genes was performed. The approach developed for the transport systems annotation does not allow this analysis to be performed because the database was small and thus the available organisms span was reduced, rendering such analysis too biased.

As a Fungus, *Kluyveromyces lactis* is expected to have a genome similar to other fungal genomes. Indeed, the homology taxonomic distribution was in accordance to the expected, because the well annotated *Saccharomyces cerevisiae* yeast was favoured by the annotation pipeline. Hence, the analysis of the taxonomic dispersion of the final annotation determined that approximately 82% of the genes identified as metabolic were *S. cerevisiae* homologues. As shown in Table 4, 1442 *K. lactis* genes were found to be homologues to a set of 1376 distinct baker’s yeast genes. There is clearly a no one-to-one relationship since, for instance, KLLA0C19338g and KLLA0D00258g were identified as homologues of the YBR093c *S. cerevisiae* gene, and annotated with the EC number 3.1.3.2. Several other *S. cerevisiae* genes were used as reference for the annotation of two or more *K. lactis* genes.

**Table 4:** **Percentage of *K. lactis *** **genes annotated as *S. cerevisiae *** **or other organisms homologues**

	**Unique**	**Total**	**%**
*K. lactis* genes with *S. cerevisiae* metabolic homologues	1376	1442	81.98%
*K. lactis* genes with other homologue organisms	39	43	2.44%
TCS families annotation	270	270	15.35%
*K. lactis* TC annotation	4	4	0.23%

The TCS families’ annotation quantifies the number of genes annotated as transporter protein encoding genes. The four TC numbers in the last row of the table were annotated by the TCDB, hence not being annotated by homology. The number of unique genes represents the number of distinct homologue genes.

*Kluyveromyces lactis*, unlike *S. cerevisiae*, did not undergo whole genome duplication [60]; nevertheless, it is likely that at least part of the 66 genes with repeated metabolic functions in *K. lactis* are a result of other gene duplication events.

The 4 genes (see Additional file 3: Table S1 of the supplemental material) reported in the transporter classification database (TCDB) were not inferred from another organism, thus not being included in the other organism’s annotation.

An example of homologues of organisms other than *S. cerevisiae* is the *LAC4* gene (KLLA0B14883g), which encodes the β-galactosidase protein (see Additional file 3: Table S7 of the supplemental material; *Escherichia coli* - 3.2.1.21) which affords *K. lactis* with the ability of converting lactose into galactose and glucose, hence being able to use lactose as sole carbon source.

The genes annotated by homology to organisms other than *S. cerevisiae* constitute less than 3% (43 genes) of the *K. lactis* genome annotated with metabolic functions. Additional file 3: Table S7 of the supplemental material lists the 25 organisms (other than *S. cerevisiae*) used for the *new annotation* of those 43 *K. lactis* genes, as well as the distinct EC numbers encoded on such genes. 5 of the 25 aforementioned organisms were of the Bacteria superkingdom. Although *K. lactis* is included in the Eukaryota superkingdom, along with the remaining 20 organisms, previous works have demonstrated the relevance of horizontal gene transfer from prokaryotic to fungal genomes [61,62].

Table 5 contains 29 genes (out of the 43 genes annotated by homology to organisms other than *S. cerevisiae*) associated with enzymes not encoded by the *S. cerevisiae* genome (according to UniProt). There were 7 other genes annotated with functions inferred from non-*Saccharomyces cerevisiae* homologue genes but whose corresponding enzymes are available in the *S. cerevisiae* genome. However, the genes that encoded such functions in the baker’s yeast did not have any homologue gene in the milk’s yeast genome. The remaining 7 non-*Saccharomyces cerevisiae* homologue genes were assigned with enzymes with partial EC numbers (e.g. KLLA0C14993g: 1.13.-.-/O74741/*Schizosaccharomyces pombe*/Eukaryota); thus, it was not possible to assess whether such functions were available on the baker’s yeast or not.

**Table 5:** ***K. lactis *** **genes which encode enzymes not available in the baker's yeast genome**

***K. lactis *** **tag**	**Homologue**	**Annotation**	**SPECIES**	**SUPERKINGDOM**	**Function**
KLLA0A02475g	Q9Y7N4	1.4.3.3	*Schizosaccharomyces pombe*	Eukaryota	D-amino-acid oxidase
KLLA0A08492g	Q99042	1.4.3.3	*Trigonopsis variabilis*	Eukaryota	D-amino-acid oxidase
KLLA0A11352g	P50167	1.1.1.250	*Scheffersomyces stipitis*	Eukaryota	D-arabinitol 2-dehydrogenase
KLLA0B00473g	P59668	1.14.19.6	*Mortierella isabellina*	Eukaryota	d-12-fatty-acid desaturase
KLLA0B04004g	Q9USY1	2.7.1.83, 3.2.-.-	*Schizosaccharomyces pombe*	Eukaryota	pseudouridine kinase, --
KLLA0B14883g	P06864	3.2.1.23	*Escherichia coli (strain K12)*	Bacteria	beta-galactosidase
KLLA0C00715g	P0A9H8	2.1.1.79	*Escherichia coli O6*	Bacteria	cyclopropane-fatty-acyl-phospholipid synthase
KLLA0C09240g	Q6SZS6	1.3.5.2	*Kluyveromyces marxianus*	Eukaryota	dihydroorotate dehydrogenase
KLLA0C11803g	O74492	3.5.2.17	*Schizosaccharomyces pombe*	Eukaryota	hydroxyisourate hydrolase
KLLA0C19107g	Q9P903	3.5.2.2	*Saccharomyces kluyveri*	Eukaryota	dihydropyrimidinase
KLLA0D00330g	P07337	3.2.1.21	*Kluyveromyces marxianus*	Eukaryota	beta-glucosidase
KLLA0D00506g	O59832	3.4.13.19	*Schizosaccharomyces pombe*	Eukaryota	membrane dipeptidase
KLLA0D03520g	Q96W94	3.5.1.6	*Saccharomyces kluyveri*	Eukaryota	beta-ureidopropionase
KLLA0D07568g	Q9Y7N4	1.4.3.3	*Schizosaccharomyces pombe*	Eukaryota	D-amino-acid oxidase
KLLA0E02641g	Q16739	2.4.1.80	*Homo sapiens*	Eukaryota	ceramide glucosyltransferase
KLLA0E10737g	Q54GH4	1.13.99.1	*Dictyostelium discoideum*	Eukaryota	inositol oxygenase
KLLA0E10935g	P78609	1.7.3.3	*Cyberlindnera jadinii*	Eukaryota	urate hydroxylase
KLLA0E14631g	P07337	3.2.1.21	*Kluyveromyces marxianus*	Eukaryota	beta-glucosidase
KLLA0E14763g	A7SMW7	1.1.99.2	*Nematostella vectensis*	Eukaryota	2-hydroxyglutarate dehydrogenase
KLLA0E15181g	Q5R778	1.3.99.3	*Pongo abelii*	Eukaryota	acyl-CoA dehydrogenase
KLLA0E18371g	Q12556	1.4.3.22	*Aspergillus niger*	Eukaryota	diamine oxidase
KLLA0E19471g	F2QNN3	1.14.19.4	*Pichia pastoris*	Eukaryota	d-8-fatty-acid desaturase
KLLA0E22397g	Q75WS0	1.1.1.184, 1.1.1.289	*Kluyveromyces aestuarii*	Eukaryota	carbonyl reductase (NADPH), sorbose reductase
KLLA0E24003g	A3GF07	1.1.1.184, 1.1.1.289	*Scheffersomyces stipitis*	Eukaryota	carbonyl reductase (NADPH), sorbose reductase
KLLA0E25081g	P07337	3.2.1.21	*Kluyveromyces marxianus*	Eukaryota	beta-glucosidase
KLLA0F03146g	P51691	3.1.6.1	*Pseudomonas aeruginosa*	Bacteria	arylsulfatase
KLLA0F04235g	Q10088	3.5.3.11	*Schizosaccharomyces pombe*	Eukaryota	agmatinase
KLLA0F07095g	P59668	1.14.19.6	*Mortierella isabellina*	Eukaryota	d-12-fatty-acid desaturase
KLLA0F27995g	O42887	3.5.3.11	*Schizosaccharomyces pombe*	Eukaryota	Agmatinase

The species name was automatically retrieved; thus, instead of the last taxonomic name, the organism name may be a synonym.

As shown in Table 5 the *Schizosaccharomyces pombe* homologue genes lead the group of functions not available in *S. cerevisae,* with five enzymes. Those enzymes were D-amino-acids oxidase (1.4.3.3), pseudouridine kinase (2.7.1.83), membrane dipeptidase (3.4.13.19), hydroxyisourate hydrolase (3.5.2.17) and agmatinase (3.5.3.11) which hydrolyses agmatine to putrescine and urea. Also, *Kluyveromyces marxianus* provides the β-glucosidase (3.2.1.21) enzyme encoding gene homologue, which releases β-D-glucose from polysaccharides containing glucose. *Mortierella isabellina* genome has a gene that encodes the δ-12 fatty acid desaturase (1.14.19.6) that catalyses the desaturation of oleic acid to linoleic acid, and *K. lactis* has two homologues of such gene (KLLA0B00473g and KLLA0F07095g). *Escherichia coli* strains have two *K. lactis* homologue genes not present in the *S. cerevisiae* genome: cyclopropane fatty acid synthase, (2.1.1.79), and the aforementioned β-galactosidase (3.2.1.23).

### Annotation scheme and manual curation results

*merlin*’s automatic scored similarity results were manually curated by the authors, using the annotation pipeline described on the methods section. The outcome of such classification is shown in Additional file 3: Table S8 of the supplemental material. It represents the results obtained using the cross classification developed and applied throughout this work. This table shows that most annotations were supported by all databases (SGD, UniProt and BRENDA), which means that the present annotation is robust and supported by information provided by several data sources.

Also, almost half (calculation details on Additional file 2) of the incorrect *merlin* automated gene annotations were reclassified by BRENDA. Most of the reclassifications dictated by BRENDA corresponded to partial EC numbers for which a complete EC number was now available in BRENDA.

BRENDA was also important for other reasons. For example, one of the *K. lactis* genes that had a baker’s yeast homologue was assigned with a completely different function in both genomes. The *XYL1* (KLLA0E21627g – 1.1.1.307) *K. lactis* gene [63] is homologue to the GRE3 (YHR104W-1.1.1.306) *S. cerevisiae* gene. However, on the first case it encodes a NADPH-dependent D-xylose reductase, but on the second organism it encodes a NADPH-dependent aldose reductase. This is a major difference because the baker’s yeast, despite having xylose transporters, cannot use xylose as the single carbon source. The *XYL1* gene is identified in UniProt [Swiss-Prot: P49378] as a “*NAD(P)H-dependent D-xylose reductase”;* yet, UniProt provides a partial EC number (1.1.1-) and KEGG annotates such gene as an hypothetical protein [KEGG: kla:KLLA0E21627g]. BRENDA was used to confirm EC number assignments, describing the reactions catalysed by those enzymes, allowing a more precise gene annotation.

Another carbon source that *S. cerevisiae* is unable to metabolise is lactose. However, in this case, the gene did not have a baker’s yeast homologue (it was an *Escherichia coli* homologue). That gene was well known to be encoded in *K. lactis*, the previously mentioned *LAC4* gene (β-galactosidase – 3.2.1.23).

Additional file 3: Table S9 of the supplemental material lists the seven genes for which literature was considered through the annotation process. The curation of those genes was based on previous knowledge of the authors regarding specificities of *Kluyveromyces lactis* metabolism.

### Assignment of enzyme commission numbers

More than 80% of the genes to which a metabolic function was assigned were classified with at least one EC number. Indeed, as shown in Table 6, 1325 (1107 + 218) genes were assigned with only one EC number (monofunctional genes). Nevertheless, three other gene groups were identified while classifying the protein encoding genes, originating 4 distinct groups:

• monofunctional genes

• multifunctional genes

• multiclass genes

• genes with EC and TC(S) numbers

**Table 6:** Enzyme encoding genes classification

		**Oxidoreductases**	**Transferases**	**Hydrolases**	**Lyases**	**Isomerases**	**Ligases**	**Total**
	**monofunctional**	165	397	347	53	44	101	1107
	**multifunctional**	13	28	8	5	1	4	59
**complete**	**multiclass**	4	4	4	3	1	2	18
	**with TC(S) number**	16	6	23	0	0	1	46
	**subtotal**	198	435	382	61	46	108	1230
	**monofunctional**	38	63	110	0	3	4	218
	**multifunctional**	0	1	2	0	0	1	4
**partial**	**multiclass**	0	4	0	0	0	0	4
	**with TC(S) number**	0	2	0	0	0	0	2
	**subtotal**	38	70	112	0	3	5	228
	**Total**	236	505	494	61	49	113	**1458**

Complete EC numbers are assigned when all classes are identified (*e.g.* 1.1.1.1). Partial EC numbers are assigned when at least one class is unknown (*e.g.* 1.1.-.- or 1.1.1.-).

The multifunctional genes set includes enzyme encoding genes that were assigned with two or more EC numbers of the same class, according to the Enzyme Commission classification (e.g. KLLA0F20163g - 2.3.1.23, 2.3.1.51). The multiclass genes encompassed enzyme encoding genes assigned with EC numbers classified in more than one class. For the last subgroup, the approach was somewhat different. The proteins may not have various functions, but had at least one EC number and one TC(S) number assigned to them. Hence, despite the distinctive classification, the function of the protein may well be the same in both classification systems.

Regardless of the previous sorting, the genes were also divided in two major categories: the ones that encoded enzymes with complete EC numbers (e.g. 1.1.1.1) and the ones that encoded enzymes with partial (e.g. 1.-.-.-) EC numbers. These two categories were then subdivided in the four sets presented above as depicted in Table 6. Thus, any gene that encoded at least one enzyme with one partial EC number was clustered with the partial entries, even for the ones that were simultaneously classified with TC(S) numbers.

Finally, the gene assignments were also cross-classified according to the EC class of the encoded proteins, those being Oxidoreductases, Transferases, Hydrolases, Lyases, Isomerases and Ligases.

The cross-classification of enzyme encoding genes assigned to the multiclass group in the EC class followed a simple rule. When classifying a gene product, such gene was assigned to the subgroup of whatever enzyme was annotated first, because such function was assumed as the main function (e.g. gene KLLA0E15357g is associated with EC numbers 6.3.5.5 and 2.1.3.2; the gene was assigned to the Ligases multiclass group instead of the Transferases multiclass group because it is assumed that the ligase function is more significant). The final result of all cross-classifications is presented in the Table 6.

As depicted in Table 6, most of the identified complete monofunctional genes encode Transferases. On the other hand, most of the genes that encode enzymes for which only a partial EC number is available are hydrolases. Table 6 also indicates that Oxidoreductases, Transferases and Hydrolases represent almost 85% of the identified enzyme encoding genes. Thus, Lyases, Isomerases and Ligases represent just a small quota of this organism’s genome.

Most enzyme encoding genes were assigned with just one EC number (1325 genes), which means that such genes are monofunctional. Still, 218 genes encoding monofunctional enzymes have only partial EC numbers assigned. Thus, either the catalysed reactions are not completely known (and therefore the enzymes may be either mono or multifunctional), or the catalysed reaction is well known but the EC number has not been assigned yet.

The multifunctional genes encode proteins that catalyse similar reactions, though using substrates with small differences, such as the case of KLLA0F20163g – (2.3.1.23, 2.3.1.51), in which

i. 1.2.3.1.23 - acyl-CoA + 1-acyl-sn-glycero-3-phosphocholine = CoA + 1,2-diacyl-sn-glycero-3-phosphocholine

ii. 2.3.1.51 - acyl-CoA + 1-acyl-sn-glycerol 3-phosphate = CoA + 1,2-diacyl-sn-glycerol 3-phosphate

These enzymes are O-acyltransferases that mediate the incorporation of unsaturated acyl chains into the sn-2 position of phospholipids.

There were also 22 genes in the *K. lactis* genome that encoded multiclass enzymes due to their diversified catalytic activity. For example, as previously mentioned, the gene KLLA0E15357g - 6.3.5.5, 2.1.3.2 encoded the homologue of the *S. cerevisiae* URA2 gene. Such protein catalyses the first two enzymatic steps in the de novo biosynthesis of pyrimidines: first L-glutamine is hydrolysed by the carbamoyl-phosphate synthase (6.3.5.5). Next, the aspartate carbamoyltransferase (2.1.3.2) uses the carbamoyl phosphate formed in the previous reaction and interacts with L-aspartate generating N-carbamoyl-L-aspartate with the release of one phosphate molecule. Hence, the gene was classified in the Ligases sub-group.

### Assignment of transporter classification numbers

Throughout this work, some enzymes encoded in the milk yeast genome were identified and classified with both EC and TC(S) numbers. In some cases, the protein was assigned with the same function by both classification systems. An example of such annotations were the functions assigned to the gene KLLA0F20658g, which encodes the Sodium transport ATPase *ENA1* (*S. cerevisiae* homologue). The protein was annotated by the enzyme commission with the EC number 3.6.3.7 (Na + exporting ATPase) and in the transporter classification database as belonging to the 3.A.3 P-type ATPase (P-ATPase) Superfamily 3.A.3.#.#, which includes proteins that promote cations, such as sodium, exchange or efflux. The transported metabolites analysis (to be published together with the transports classification methodology in Genome-wide semi-automated annotation of transporter systems, Dias *et al.*, 2012), provided by *merlin*, confirms that such gene facilitates the efflux of sodium ions, among the transport of other cations.

**Figure 5:**
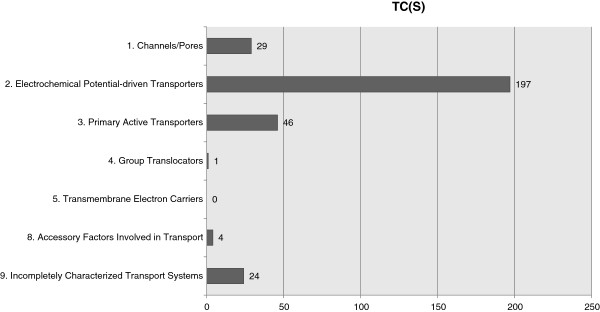
TC(S) numbers distribution.

In this work, 301 genes were assigned with, at least, one TC(S) number and no EC number, which means that such genes are exclusively associated to transport mechanisms. As depicted in Figure 5, more than 65% of the transporter proteins (without EC numbers) identified in this work were electrochemical potential-driven transporters. The electrochemical potential-driven transporters class encompasses several protein families, such as sugar and monocarboxylate porters, drugs and UDP-Galactose:UMP antiporters, amino acids and chloride transporters, zinc and iron permeases or cation and phosphate symporters, among many others [50]. According to Additional file 3: Table S10 of the supplemental material, although all 199 *K. lactis* genes classified as electrochemical potential-driven transporters belong to the 2.A Porters (uniporters, symporters, antiporters) sub-class, such genes are classified in 63 distinct families. Nevertheless, most of the Porters identified within the *K. lactis* genome (75 genes) were carriers of the 2.A.1 – Major Facilitator Superfamily (MFS). According to Law *et al*. (2008) [64] the MFS encompasses proteins that transport several substrates through an energy independent carrier mediated process, binding the transporter to the solute and undergoing a series of conformational changes. Such superfamily includes the secondary active membrane transporters, which sort the transporters through the kinetic mechanism, used to carry the substrate, in three categories: uniporters, symporters and antiporters [55,64].

The classification of two thirds of the transport systems available in *K. lactis* in the Porters sub-class suggests that this microorganism may be able to control the uptake and efflux of the nutrients, providing the organism with the ability to be selective about the carbon source it will use.

Additional file 3: Table S10 of the supplemental material also demonstrates that at least 21 broad sugar porters encoding genes were identified, as well as several alcohols, organic acids and nitrogen sources and amino acid transport systems. It is accepted that non-ionized organic acids can penetrate cell walls by passive diffusion [65]. Thus, evidences of organic acids transport systems may be related to the transport of ionized organic acids and with the need for controlling the uptake or excretion of those compounds.

Furthermore, *Kluyveromyces lactis* can use several alcohols as carbon sources, as demonstrated in [66-71]. Some of those alcohols are known as sugar alcohols (polyols) and are transported by the sugar porter family transport systems 2.A.1.1.# encoded in genes KLLA0E06755g and KLLA0E01783g. Three glycerol transport systems were also identified during the course of this work (KLLA0A03223g - 2.A.1.1.#, KLLA0F26246g - 2.A.1.1.#, KLLA0E19185g - 2.A.50.1.#, KLLA0E00617g - 1.A.8.5.#).

### KEGG pathways annotation analysis

Table 7 demonstrates that the *new annotation* identified several new enzymes in global pathways. Global pathways are universal, and include enzymes from several pathways, which may or may not be available in *K. lactis*. Thus, Additional file 3: Table S11 of the supplemental material depicts the pathways in which new enzymes have been identified in the *new annotation*, as well as the number of unidentified enzymes, and the enzymes identified by both the *new annotation* and KEGG.

**Table 7:** Number of enzymes in each Global pathway

**Global pathways**	**Identified by KEGG**	**Identified in *new annotation***	**Unidentified in *K. lactis***	**Total**
01100 Metabolic pathways	383	51	869	1303
01110 Biosynthesis of secondary metabolites	167	12	349	528
01120 Microbial metabolism in diverse environments	93	8	351	452

The *new annotation* provides new insights on the *K. lactis* metabolic capabilities, as it brings new information to the KEGG pathways, identifying several new enzymes in 56 KEGG metabolic pathways. Indeed, only 45 of such pathways are recognised by KEGG as *K. lactis* pathways. Thus, the other 11 pathways should be further studied to assess if the milk yeasts uses such paths to metabolise compounds, offering investigators new research opportunities.

Nevertheless, the *new annotation* also identified new enzymes that are not allocated to any pathway and proteins associated only with TC numbers.

### Analysis of the Annotation of the *Kluyveromyces lactis* central carbon metabolism

The central carbon metabolism is a collection of pathways mainly composed by three *‘vias’*, namely the Embden-Meyerhof-Parnas (EMP) Pathway, the Pentose Phosphate Pathway and the TCA Cycle. The *new annotation* presented in this work was able to identify the genes involved in such pathways.

The EMP pathway converts glucose to pyruvate, generating small amounts of ATP and NADH in the process. The uptake of glucose is done by hexose transporters such as *RAG1* – KLLA0D13310g, *HGT1* – KLLA0A11110g, *KHT1* or *KHT2*. In some strains *RAG1* is the unique low-affinity glucose transporter, whereas in other strains such function is divided by two genes (*KHT1*, *KHT2*). The strain studied throughout this work, *Kluyveromyces lactis* NRRL Y-1140 (CBS 2359), encoded the *RAG1* gene.

The EMP pathway has only one hexokinase, *RAG5* (KLLA0D11352g) which was identified in the *new annotation*. Breunig and Steensma (2003) [72] confirm that it is the only hexokinase encoding gene, unlike in the case of *S. cerevisiae*, which has three hexokinases. *RAG5* is an essential gene because its absence inhibits growth on glucose, fructose and higher sugars that produce these isomers. Glucose-6-phosphate isomerase *RAG2* (KLLA0E23519g) was also identified in the *new annotation*, and, although *K. lactis* has only one phosphoglucose isomerase, *RAG2* mutants grow well in glucose. Hence, *RAG2* is not an essential gene.

**Figure 6:**
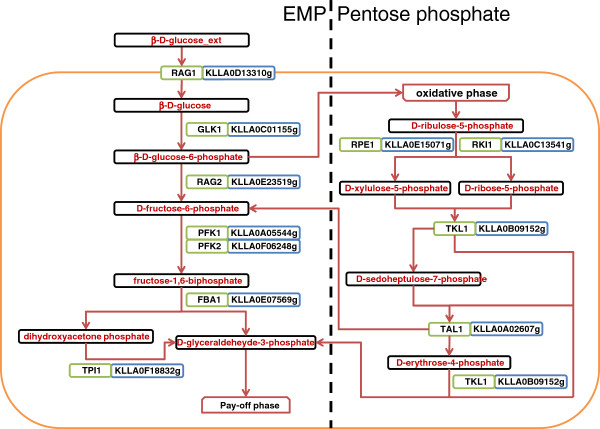
**EMP and Pentose Phosphate pathways after the *new annotation.*** Enzymes (green), gene identifiers (blue) and metabolites (red).

The oxidative phase of the pentose phosphate pathway generates 2 NADPH molecules from the conversion of glucose-6-phosphate into ribulose 5-phosphate which is then delivered to the non-oxidative phase (Figure 6). If either the 6 – phosphofructokinase protein complex encoding genes or the triosephosphate isomerase encoding gene *TPI1* (KLLA0F18832g) are deleted, the transaldolase *TAL1* (KLLA0A02607g), together with the transketolase *TKL1* (KLLA0B09152g) can convert ribulose 5 – phosphate into fructose 6 – phosphate D-glyceraldehyde 3-phosphate and surpass the phosphofructokinase complex deletion, as depicted in Figure 5.

Several ATP and NADH molecules are formed in the second half of glycolysis, which is known as the pay-off phase. There are NADPH dehydrogenases not present in the baker’s yeast reported to exist in the milk yeast genome. Such enzymes are *NDE1* (KLLA0E21891g) and *NDE2* (KLLA0A08316g), and were indeed annotated in the present work. Both genes re-oxidise NADH as well as NADPH. *NDE1*’s ability to bind NADPH was verified experimentally [73]. However, *NDE2* was reported to have a less important role in NADPH re-oxidation [74]. *NDI1* (KLLA0C06336g) also encodes a mitochondrial internal NADH oxidoreductase, though such enzyme does not oxidise NADPH. Neither *NDE1*, *NDE2* or *NDI1* are annotated in UniProt and are incorrectly annotated in KEGG.

The re-oxidation of NAD(P)H by mitochondrial external dehydrogenases supports the high activity of the pentose phosphate pathway, and the ability of the *K. lactis RAG2* mutants to grow on glucose.

In Crabtree negative yeasts, such as *K. lactis*, ethanol formation only sets in when the oxygen supply becomes limiting. According to Van Urk *et al*. (1989) [75], Crabtree negative yeasts can prevent the overflow metabolism, by regulating the glucose uptake using the available symport transport mechanisms to control the amount of glucose going inside the cells. The *new annotation* provided by this work demonstrates that more than 65% of the identified transport systems were classified in the 2.A – Porters (uniporters, symporters, antiporters), allowing *K. lactis* to regulate nutrients uptake and efflux. However, Breunig and Steensma state that the regulation of the glucose uptake is not enough to explain the Crabtree negative phenotypes. Only when the pyruvate dehydrogenase (Pdh) complex is down regulated, or blocked, the pyruvate decarboxylase (Pdc) can convert pyruvate to ethanol and acetaldehyde [1]. The first step of the alcoholic fermentation, which only occurs at low oxygen concentrations, is promoted by the pyruvate decarboxylase *PDC1* (identified in gene KLLA0E16303g). Therefore, a null mutation on the *PDA1* (KLLA0F12001g), a gene which encodes the α subunit of the E1 component (the β subunit was identified in the *new annotation,* gene *PDB1*_KLULA – KLLA0F09603g, not identified in UniProt) of the Pdh complex, can constrain growth on glucose, as *PDA1* mutants show high ethanol formation [76]. Such phenotype suggests that high Pdh activity is the reason for the Crabtree negative phenotype exhibited by the wild type strain.

The lactose metabolism in *Kluyveromyces lactis* has been well studied, because it is a distinct characteristic within yeasts. The lactose uptake is performed by the specific permease *LAC12* (KLLA0B14861g) and the hydrolysis by the β–galactosidade *LAC4* (KLLA0B14883g) into glucose and galactose. The lactose metabolism is induced by both lactose and galactose. Galactose is converted into galactose–1–phosphate by galactokinase *GAL1* (KLLA0F08393g). Then, the *GAL7* (KLLA0F08437g) gene that encodes the enzyme UDP-glucose-hexose-1-phosphate uridylyltransferase takes UDP–glucose and α–D–galactose–1–phosphate to synthesize α–D-glucose–1–phosphate and UDP–galactose. The UDP–galactose formed by this reaction will be again converted to UDP–glucose by the *GAL10* bifunctional gene. This gene encodes two enzymes, the aforementioned UDP–glucose–4–epimerase and the aldose–1–epimerase, that converts α–D–glucose into β–D–glucose. All of the genes described earlier were annotated throughout this work.

### Assessing the agreement of the *new annotation* to a previous comparison of the *Kluyveromyces lactis* genome to the one of *Saccharomyces cerevisiae*

In 1998 Ozier-Kalogeropoulos *et al*. [77] studied the *Kluyveromyces lactis* unsequenced genome, and identified 296 *K. lactis* genes with homology to the baker’s yeast. The exploration of the genome was random, thus several types of genes were identified.

All *S. cerevisiae* genes identified in that study were reviewed in UniProt (SGD does not provide an application programming interface to expedite the results retrieval) to identify genes with metabolic (enzymatic or transport) functions. As depicted in Additional file 3: Table S12 of the supplemental material, 113 of those *S. cerevisiae* genes had metabolic functions. The 113 metabolic genes identified in that study, and the corresponding milk yeast homologues, were predicted by the *new annotation*, except for four baker’s yeast transport systems which were not identified because the corresponding *K. lactis* homologues did not have any transmembrane domain.

Again, that work was in agreement with the results obtained with the approach undertaken throughout this study.

In conclusion, these examples illustrate that the *new annotation* not only confirms pre-sequencing knowledge but also, adds new gene annotations to the information currently available in databases such as KEGG or UniProt.

## Conclusions

Since the genome sequence of *K. lactis* was published in 2004, the proteins encoded in the *Kluyveromyces lactis* genome had never been thoroughly reviewed and annotated; or at least this information was not published, to our knowledge.

In this work, 2000 genes with potential to be assigned with metabolic functions within the proteins encoded in the *Kluyveromyces lactis* genome were studied. Most of those, specifically 87.95% (1759 genes), were indeed classified as metabolic genes. The metabolic genes could be exclusively enzymatic (1410 genes), transporter proteins (301 genes) or have both metabolic activities (48 genes). The *new annotation* proposed in this work could only be accomplished as *merlin* provided semi-automatic scored results. Such results were then reviewed in other databases such as UniProt or BRENDA to maximize the confidence in the results. The *new annotation* includes novelties, such as the assignment of transporter superfamily numbers to genes identified as transporter proteins. Moreover, it was demonstrated that Oxidoreductases, Transferases and Hydrolases represent almost 85% of the identified enzymes. When the *new annotation* is compared to the annotations currently available in some databases, it is shown to be broader and reliable, as it encompasses most of the metabolic information in such databases.

Furthermore, the *new annotation* of the *K. lactis* metabolic genome confirmed the predictions of pre-genome sequencing studies. One of those studies compared random sequences of the *K. lactis* genome to the *S. cerevisiae* sequenced genome. All metabolic genes in that study were identified in the *new annotation*.

Also, the central carbon pathways were revised in this work to assess the robustness of the *new annotation*. The *new annotation* was in agreement with several publications that study *Kluyveromyces lactis’* phenotypical behaviour.

The *new annotation* provided by this study, available in Additional file 4 on the GenBank format, yields basic knowledge which might be useful for the scientific community working on this model yeast, as new functions have been identified for the so-called metabolic genes.

The methodology used throughout this work can be used by other groups to annotate other organisms and build a robust genome-scale model.

Furthermore, the *new annotation* served as the basis for the reconstruction of a compartmentalized, genome-scale metabolic model of *Kluyveromyces lactis*, which is currently being finished.

## Abbreviations

BLAST: Basic local alignment search tool; BRENDA: BRaunschweig ENzyme DAtabase; EC: Enzyme commission; EEGC: enzyme encoding gene candidate; IUBMB: International union of biochemistry and molecular biology; *Merlin*: MEtabolic models reconstruction using genome-scaLe information; NCBI: National center for biotechnology information; *nrDB*: All non-redundant sequences; PDB: Brookhaven protein data; PIR: Protein information resource; PRF: Protein research foundation; RefSeq: Reference sequences; SGD: Saccharomyces genome database; TCDB: Transporter classification database; TC: Transporter classification; TCS: Transporter classification superfamily; TPGC: Transporter protein encoding gene candidates; *yeastDB*: yeast database; UniProt: Universal protein resource.

## Competing interests

The author(s) declare that they have no competing interests.

## Authors’ contributions

OD carried out the annotation and drafted the manuscript. ECF participated in the design of the study and helped to draft the manuscript. IR and AKG conceived the study, participated in its design and coordination and helped to draft the manuscript. All authors read and approved the final manuscript.

## Supplementary Material

Additional file 1 **Figure S1.** Merlin’s annotation interface. Figure S2. blast output format. Click here for file

Additional file 2 Classification of Manual Curation Results. Click here for file

Additional file 3 **Table S1.** K. lactis genes TCDB annotation. Table S2. Final annotation of the 2000 genes reviewed in this publication. Table S3. Comparison between the new annotation and KEGG’s annotation. Table S4. List of genes annotated as metabolic by KEGG but ruled out as metabolic in the new annotation. Table S5. Comparison between the new annotation and UniProt’s annotation. Table S6. Comparison between the new annotation and BRENDAS’s annotation. Table S7. List of genes assigned with metabolic functions by homology to organisms other that Saccharomyces cerevisiae in the new annotation. Table S8. Alphanumeric homologues classification. Table S9. Genes annotated using literature as main source. Table S10. List of genes assigned with transport functions, corresponding Superfamilies’ and families numbers. Table S11. KEGG Pathways in which new enzymes have been identified, by the new annotation. Table S12. Comparison between Ozier-Kalogeropoulos et.al. (1998) and the new annotation. Click here for file
