# Supplementary material for: Genome-wide metabolic (re-) annotation of Kluyveromyces lactis
Source: BMC Genomics. 2012 Oct 1;13:517. doi: 10.1186/1471-2164-13-517 (PMC3508617; doi:10.1186/1471-2164-13-517)
Supplement: Additional file 1 — Figure S1. Merlin’s annotation interface. Figure S2. blast output format. [file 1471-2164-13-517-S1.pdf]

summary

- **Figure S1.** *merlin*'s annotation interface.
- **Figure S2.** blast output format.

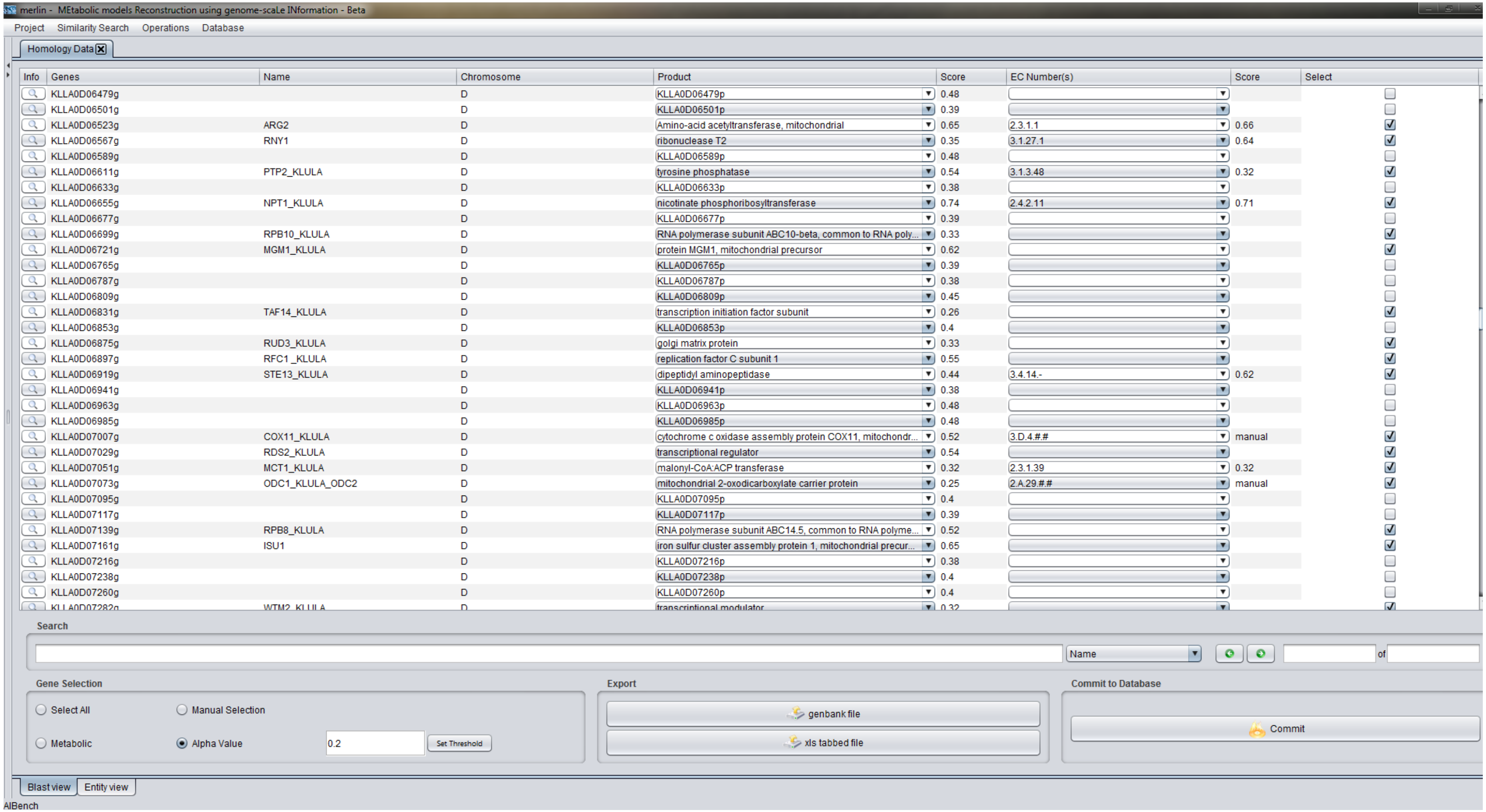

merlin - MEtabolic models Reconstruction using genome-scaLe INformation - Beta
Project Similarity Search Operations Database
Homology Data
Info Genes Name Chromosome Product Score EC Number(s) Score Select
KLLA0D06479g D KLLA0D06479p 0.48
KLLA0D06501g D KLLA0D06501p 0.39
KLLA0D06523g ARG2 D Amino-acid acetyltransferase, mitochondrial 0.65 2.3.1.1 0.66
KLLA0D06567g RNY1 D ribonuclease T2 0.35 3.1.27.1 0.64
KLLA0D06589g D KLLA0D06589p 0.48
KLLA0D06611g PTP2_KLULA D tyrosine phosphatase 0.54 3.1.3.48 0.32
KLLA0D06633g D KLLA0D06633p 0.38
KLLA0D06655g NPT1_KLULA D nicotinate phosphoribosyltransferase 0.74 2.4.2.11 0.71
KLLA0D06677g D KLLA0D06677p 0.39
KLLA0D06699g RPB10_KLULA D RNA polymerase subunit ABC10-beta, common to RNA poly... 0.33
KLLA0D06721g MGM1_KLULA D protein MGM1, mitochondrial precursor 0.62
KLLA0D06765g D KLLA0D06765p 0.39
KLLA0D06787g D KLLA0D06787p 0.38
KLLA0D06809g D KLLA0D06809p 0.45
KLLA0D06831g TAF14_KLULA D transcription initiation factor subunit 0.26
KLLA0D06853g D KLLA0D06853p 0.4
KLLA0D06875g RUD3_KLULA D golgi matrix protein 0.33
KLLA0D06897g RFC1_KLULA D replication factor C subunit 1 0.55
KLLA0D06919g STE13_KLULA D dipeptidyl aminopeptidase 0.44 3.4.14.- 0.62
KLLA0D06941g D KLLA0D06941p 0.38
KLLA0D06963g D KLLA0D06963p 0.48
KLLA0D06985g D KLLA0D06985p 0.48
KLLA0D07007g COX11_KLULA D cytochrome c oxidase assembly protein COX11, mitochondr... 0.52 3.D.4.#.# manual
KLLA0D07029g RDS2_KLULA D transcriptional regulator 0.54
KLLA0D07051g MCT1_KLULA D malonyl-CoA:ACP transferase 0.32 2.3.1.39 0.32
KLLA0D07073g ODC1_KLULA_ODC2 D mitochondrial 2-oxodicarboxylate carrier protein 0.25 2.A.29.#.# manual
KLLA0D07095g D KLLA0D07095p 0.4
KLLA0D07117g D KLLA0D07117p 0.39
KLLA0D07139g RPB8_KLULA D RNA polymerase subunit ABC14.5, common to RNA polyme... 0.52
KLLA0D07161g ISU1 D iron sulfur cluster assembly protein 1, mitochondrial precur... 0.65
KLLA0D07216g D KLLA0D07216p 0.38
KLLA0D07238g D KLLA0D07238p 0.4
KLLA0D07260g D KLLA0D07260p 0.4
KLLA0D07282g WTM2_KLULA D transcriptional modulator 0.32
Search
Name
of
Gene Selection
Select All
Manual Selection
Metabolic
Alpha Value
0.2
Set Threshold
Export
genbank file
xls tabbed file
Commit to Database
Commit
Blast view
Entity view
AIBench

```
BLASTP 2.2.22+
Reference: Stephen F. Altschul, Thomas L. Madden, Alejandro
A. Schaffer, Jinghui Zhang, Zheng Zhang, Webb Miller, and
David J. Lipman (1997), "Gapped BLAST and PSI-BLAST: a new
generation of protein database search programs", Nucleic
Acids Res. 25:3389-3402.


RID: HMNCS29H013


Database: All non-redundant GenBank CDS
translations+PDB+SwissProt+PIR+PRF excluding environmental samples
from WGS projects
           10,140,583 sequences; 3,459,212,347 total letters
Query=  gi|49640135|emb|CAH02592.1| KLLA0A00110p [Kluyveromyces lactis]
Length=348


                                                                  Score     E
Sequences producing significant alignments:                       (Bits)  Value

ref|XP_451004.1|  unnamed protein product [Kluyveromyces lacti...   718    0.0
ref|XP_452199.1|  unnamed protein product [Kluyveromyces lacti...   718    0.0
ref|XP_451534.1|  unnamed protein product [Kluyveromyces lacti...   403    1e-110
ref|XP_452496.1|  unnamed protein product [Kluyveromyces lacti...   390    9e-107
ref|XP_453642.1|  unnamed protein product [Kluyveromyces lacti...   358    5e-97
ref|XP_002556506.1|  KLTH0H14982p [Lachancea thermotolerans] >...   327    1e-87
ref|XP_002497331.1|  ZYRO0F03058p [Zygosaccharomyces rouxii] >...   288    8e-76
ref|XP_001524555.1|  conserved hypothetical protein [Lodderomy...   217    1e-54
```
