# Supplementary material for: Genome-wide metabolic (re-) annotation of Kluyveromyces lactis
Source: BMC Genomics. 2012 Oct 1;13:517. doi: 10.1186/1471-2164-13-517 (PMC3508617; doi:10.1186/1471-2164-13-517)
Supplement: Additional file 2 — Classification of Manual Curation Results. [file 1471-2164-13-517-S2.pdf]

Classification of Manual Curation Results

When using the annotation pipeline to analyse the EEGC's, a limited number of logical jumps were detected. Thus, an alpha-numeric cross classification system was developed to log and identify the genes classification patterns.

Such classification encompassed two features. The first concerned the origin of the entry chosen in the final annotation. Numbers represent the alignment and matching results for the two previously described *merlin* projects. The available options were:

1 – the *nrDB* entry was considered the correct one and was therefore chosen;

2 – the *yeastDB* entry was chosen;

3 - both entries were considered correct although they were distinct (merge entries);

4 - both were considered incorrect (the EEGC was annotated with an EC number by other database);

5 - *nrDB* entry was chosen but is incomplete;

6 - *yeastDB* entry was chosen but is incomplete;

7 - no encumber.

The second factor was the database(s) that provided the information that motivated the choice made. Such factor was expressed by selecting one of the following alternatives:

a – SGD, UniProt and BRENDA (meaning that all these three databases had similar information);

b - SGD and UniProt;

c - SGD and BRENDA;

d - UniProt and BRENDA;

e – SGD;

f – UniProt;

g – BRENDA;

h – TCDB;

l – literature;

x – no data.

Additionally, in some cases extra information was used for the genes that were already annotated in UniProt as *K. lactis*' reviewed records. In these cases, an α was used to differentiate them. Moreover, the candidate gene records that were annotated as homologues of organisms other than *S. cerevisiae* have a β to distinguish them. Such features were concatenated with the previously described numeric classification.

For example, classifying a gene as *1f* means that such gene entry was annotated as described on the first project (provided by the *nrDB*) and such information was confirmed by accessing the *S. cerevisiae* record on UniProt. If a gene had incomplete entries on both projects then it would be classified as *5/6a*, as long as the record annotation was confirmed by SGD, UniProt and BRENDA. If a gene is cross classified with *2αa* it means that such gene was correctly annotated on the second project (2), and the assignment was confirmed by querying all three databases (SGD, UniProt and BRENDA) on the assignment of the *S. cerevisiae* homologue (*a*) and, moreover, UniProt had already annotated such *Kluyveromyces lactis* gene with a reviewed record (*α*).

The established cross classification was used to evaluate each of the collections obtained by lining up both merlin projects (matches, *nrDB* assigned, distinct, *yeastDB* assigned), as well as the previously identified transporter protein encoding gene.

The first line-up cluster (matches) encompasses EEGC's that were assigned with the same function on both similarity searches (*nrDB* and *yeastDB*), thus by both merlin projects. Therefore, a simplification was performed when applying the alpha-numeric

cross classification system to such set. If the assignment was considered correct, the EEGC annotation would be classified as 1 instead of being classified as both 1 (*nrDB* entry is the correct one) and 2 (*yeastDB* entry is the correct one). Such EEGC's could not have been classified as 3 (both are correct (merge entries)) because such classification is only used for cases where both projects assign correct yet distinct annotations, thus forcing the merge of both annotations. For the other three groups (*nrDB* assigned, distinct and *yeastDB* assigned) no simplifications were performed.

Annotation Schema and Manual Curation Results

merlin's automatic scored similarity results were manually curated, using the annotation pipeline described on the methods section. The outcome of such classification is shown in Additional file 3, table S8 of the supplemental material. It represents the results obtained using the cross classification developed and applied throughout this work. The numbers on the "y-axis" of the table identify the EEGC as *S. cerevisiae* or other organism homologues and such homology is confirmed in the databases represented by letters on the "x axis", as described on the methods section.

Such table shows that most annotations were confirmed, or refuted, by all databases (SGD, UniProt and BRENDA), which means that the new annotation is robust and supported by information provided by several data sources. Most of the annotated genes were assigned with the same function on both annotation projects, (*matches*). The second set of genes, the *nrDB exclusive assignments*, encompasses 466 EEGC's of which 241 (adding 7+7α+7β of the *nrDB* section of the table) were wrongly associated to enzymes (false positives). Still, 28 of those incorrectly annotated genes (7h+7αh) were actually transporter proteins assigned with TC numbers. The *distinct* set of genes represents only a little over 7% (146 EEGC's) of the total annotation (of which 18 annotations were considered false positives).

The last set of genes (*yeastDB exclusive assignments*) was the smallest one with 8 EEGC's (2 false positives) for subsequent analysis.

Such table also included the transporters annotation results, so that the approach developed to identify transport systems could be compared, in absolute values, to the approach developed for the EC numbers assignment. The transporter proteins encoding genes that were not simultaneously identified as EEGC's by the BLAST similarity searches were accounted for in the TC(S) section.

Also, more than 75% of the genes present in UniProt as *K. lactis* reviewed entries were identified in the matches cluster. Such results are due to the fact that the *K. lactis* reviewed entries in UniProt were recurrently available for genes with high similarity to other organisms.

Almost half (103 over 251 obtained by adding all values on rows 4, 4α and 4β) of the incorrect *merlin* automated gene annotations were reclassified by BRENDA (4g, 4αg, 4βg). Most of the reclassifications dictated by BRENDA corresponded to partial EC numbers for which a complete EC number was now available in BRENDA.

However, BRENDA was also important for other reasons. For example, one of the *K. lactis* genes that had a baker's yeast homologue was assigned with a completely different function in both genomes. The *XYL1* (KLLA0E21627g – 1.1.1.307) *K. lactis* gene is homologue to the *GRE3* (YHR104W-1.1.1.306) *S. cerevisiae* gene. However, on the first case it encodes a NADPH-dependent D-xylose reductase, but on the second organism it encodes a NADPH-dependent aldose reductase. This is a major difference because the baker's yeast, despite having xylose transporters, cannot use xylose as the single carbon source. BRENDA was used to confirm EC number assignments, by describing the reactions catalysed by such enzymes, allowing a more precise gene annotation. Another carbon source that *S. cerevisiae* is unable to metabolise is lactose.

However, in this case, the gene did not have a baker's yeast homologue (it was an *Escherichia coli* homologue). That gene was well known to be encoded in *K. lactis,* the *LAC4* gene (β-galactosidase – 3.2.1.23).
